# Intergenerational transmission of the effects of maternal exposure to childhood maltreatment in the USA: a retrospective cohort study

**DOI:** 10.1016/S2468-2667(23)00025-7

**Published:** 2023-03

**Authors:** Nora K Moog, Peter D Cummings, Kathryn L Jackson, Judy L Aschner, Emily S Barrett, Theresa M Bastain, Courtney K Blackwell, Michelle Bosquet Enlow, Carrie V Breton, Nicole R Bush, Sean C L Deoni, Cristiane S Duarte, Assiamira Ferrara, Torie L Grant, Alison E Hipwell, Kathryn Jones, Leslie D Leve, Stephanie Lovinsky-Desir, Richard K Miller, Catherine Monk, Emily Oken, Jonathan Posner, Rebecca J Schmidt, Rosalind J Wright, Sonja Entringer, Hyagriv N Simhan, Pathik D Wadhwa, Thomas G O’Connor, Rashelle J Musci, Claudia Buss PhD

**Affiliations:** Institute of Medical Psychology, Charité–Universitätsmedizin Berlin, corporate member of Freie Universität Berlin and Humboldt–Universität zu Berlin, Berlin, Germany; Department of Medical Social Sciences, Feinberg School of Medicine, Northwestern University, Chicago, IL, USA; Department of Medical Social Sciences, Feinberg School of Medicine, Northwestern University, Chicago, IL, USA; Department of Pediatrics, Hackensack Meridian School of Medicine, Nutley, NJ, USA; Albert Einstein College of Medicine, Bronx, NY, USA; Department of Biostatistics and Epidemiology, Rutgers School of Public Health, Environmental and Occupational Health Sciences Institute, Piscataway, NJ, USA; Department of Population and Public Health Sciences, Keck School of Medicine, University of Southern California, Los Angeles, CA, USA; Department of Medical Social Sciences, Feinberg School of Medicine, Northwestern University, Chicago, IL, USA; Department of Psychiatry and Behavioral Sciences, Boston Children’s Hospital, Boston, MA, USA; Department of Psychiatry, Harvard Medical School, Boston, MA, USA; Department of Preventive Medicine, University of Southern California, Los Angeles, CA, USA; Department of Psychiatry and Behavioral Sciences and Department of Pediatrics, Division of Developmental Medicine, Weill Institute for Neurosciences, University of California, San Francisco, San Francisco, CA, USA; Advanced Baby Imaging Lab, School of Engineering, Brown University, Providence, RI, USA; Department of Psychiatry, New York State Psychiatric Institute, Columbia University, New York, NY, USA; Division of Research, Kaiser Permanente Northern California, Oakland, CA, USA; Department of Pediatrics, Johns Hopkins University School of Medicine, Baltimore, MD, USA; Department of Psychiatry and Department of Psychology, University of Pittsburgh, Pittsburgh, PA, USA; Department of Population Medicine, Harvard Pilgrim Health Care Institute, Harvard Medical School, Boston, MA, USA; Prevention Science Institute, University of Oregon, Eugene, OR, USA; Department of Pediatrics, College of Physicians and Surgeons, Columbia University, New York, NY, USA; Department of Obstetrics and Gynecology, School of Medicine and Dentistry, University of Rochester, Rochester, NY, USA; Department of Pediatrics, School of Medicine and Dentistry, University of Rochester, Rochester, NY, USA; Department of Psychiatry, New York State Psychiatric Institute, Columbia University, New York, NY, USA; Department of Population Medicine, Harvard Pilgrim Health Care Institute, Harvard Medical School, Boston, MA, USA; Department of Psychiatry and Behavioral Sciences, Duke University School of Medicine, Durham, NC, USA; Department of Public Health Sciences and the MIND Institute, School of Medicine, University of California Davis, Sacramento, CA, USA; Department of Pediatrics, Institute for Exposomic Research, Icahn School of Medicine at Mount Sinai, New York, NY, USA; Institute of Medical Psychology, Charité–Universitätsmedizin Berlin, corporate member of Freie Universität Berlin and Humboldt–Universität zu Berlin, Berlin, Germany; Department of Pediatrics, School of Medicine, University of California, Irvine, Orange, CA, USA; Department of Obstetrics, Gynecology, and Reproductive Sciences, Magee Women’s Hospital, University of Pittsburgh, Pittsburgh, PA, USA; Department of Pediatrics, School of Medicine, University of California, Irvine, Orange, CA, USA; Department of Psychiatry and Human Behavior, University of California, Irvine, Orange, CA, USA; Department of Epidemiology, University of California, Irvine, Orange, CA, USA; Department of Obstetrics and Gynecology, School of Medicine and Dentistry, University of Rochester, Rochester, NY, USA; Department of Pediatrics, School of Medicine and Dentistry, University of Rochester, Rochester, NY, USA; Department of Psychiatry, Psychology, and Neuroscience, School of Medicine and Dentistry, University of Rochester, Rochester, NY, USA; Department of Mental Health, Johns Hopkins University School of Medicine, Baltimore, MD, USA; Institute of Medical Psychology, Charité–Universitätsmedizin Berlin, corporate member of Freie Universität Berlin and Humboldt–Universität zu Berlin, Berlin, Germany; Department of Pediatrics, School of Medicine, University of California, Irvine, Orange, CA, USA

## Abstract

**Background:**

Childhood maltreatment is associated with adverse health outcomes and this risk can be transmitted to the next generation. We aimed to investigate the association between exposure to maternal childhood maltreatment and common childhood physical and mental health problems, neurodevelopmental disorders, and related comorbidity patterns in offspring.

**Methods.:**

We conducted a retrospective cohort study using data from the Environmental influences on Child Health Outcomes (ECHO) Program, which was launched to investigate the influence of early life exposures on child health and development in 69 cohorts across the USA. Eligible mother–child dyads were those with available data on maternal childhood maltreatment exposure and at least one child health outcome measure (autism spectrum disorder, attention-deficit hyperactivity disorder [ADHD], internalising problems, obesity, allergy, and asthma diagnoses). Maternal history of childhood maltreatment was obtained retrospectively from the Adverse Childhood Experiences or Life Stressor Checklist questionnaires. We derived the prevalence of the specified child health outcome measures in offspring across childhood and adolescence by harmonising caregiver reports and other relevant sources (such as medical records) across cohorts. Child internalising symptoms were assessed using the Child Behavior Checklist. Associations between maternal childhood maltreatment and childhood health outcomes were measured using a series of mixed-effects logistic regression models. Covariates included child sex (male or female), race, and ethnicity; maternal and paternal age; maternal education; combined annual household income; maternal diagnosis of depression, asthma, ADHD, allergy, or autism spectrum disorder; and maternal obesity. Two latent class analyses were conducted: to characterise patterns of comorbidity of child health outcomes; and to characterise patterns of co-occurrence of childhood maltreatment subtypes. We then investigated the association between latent class membership and maternal childhood maltreatment and child health outcomes, respectively.

**Findings:**

Our sample included 4337 mother–child dyads from 21 longitudinal cohorts (with data collection initiated between 1999 and 2016). Of 3954 mothers in the study, 1742 (44%) had experienced exposure to abuse or neglect during their childhood. After adjustment for confounding, mothers who experienced childhood maltreatment were more likely to have children with internalising problems in the clinical range (odds ratio [OR] 2·70 [95% CI 1·95–3–72], p<0·0001), autism spectrum disorder (1·70 [1·13–2·55], p=0·01), ADHD (2·09 [1·63–2·67], p<0–0001), and asthma (1·54 [1·34–1·77], p<0·0001). In female offspring, maternal childhood maltreatment was associated with a higher prevalence of obesity (1·69 [1·17–2·44], p=0·005). Children of mothers exposed to childhood maltreatment were more likely to exhibit a diagnostic pattern characterised by higher risk for multimorbidity. Exposure to multiple forms of maltreatment across all subtypes of maternal childhood maltreatment was associated with the highest risk increases for most offspring health outcomes, suggesting a dose–response relationship.

**Interpretation:**

Our findings suggest that maternal childhood maltreatment experiences can be a risk factor for disease susceptibility in offspring across a variety of outcomes and emphasise the need for policies focusing on breaking the intergenerational transmission of adversity.

**Funding:**

Environmental influences on Child Health Outcomes Program, Office of the Director, National Institutes of Health.

## Introduction

Large, population-based retrospective surveys in the USA and Europe suggest that approximately a third of children experience some form of childhood maltreatment.^1,2^ Globally, lifetime prevalence of maltreatment experiences during childhood and adolescence varies between 8% and 35%, depending on maltreatment type and child sex.^3^ Childhood maltreatment encompasses all acts of abuse or neglect by a parent or caregiver that result in physical or emotional harm, potential for harm, or threat of harm to a child^4^ and is considered one of the most important preventable contributors to poor health outcomes later in life.^5,6^ The well established sequelae of childhood maltreatment include psychiatric disorders, including depression, anxiety, post-traumatic stress disorder, and substance abuse disorders, in addition to chronic physical conditions, including cardiovascular disease, respiratory illnesses, obesity, diabetes, and autoimmune diseases.^7,8^

A growing body of evidence suggests that the detrimental outcomes associated with childhood maltreatment might extend beyond individuals who are directly exposed, affecting the next generation,^7,8^ and thus multiplying the number of affected individuals and the social and economic impact of the exposure. The intergenerational transmission of disease susceptibility due to childhood maltreatment has been investigated mainly along the maternal line of transmission, with individual studies indicating an increased risk of mental health problems in children of mothers exposed to childhood maltreatment, including internalising problems (primarily depressive and anxiety symptoms) and externalising problems (conduct problems and hyperactivity); neurodevelopmental disorders, including autism spectrum disorder and attention-deficit hyperactivity disorder (ADHD); and physical health problems and risk factors, including asthma, allergy, and obesity.^7,8^ Most studies have focused on a single aspect of physical or mental health, despite the well established and considerable comorbidity among psychiatric and somatic conditions.^9,10^ As a result, whether maternal childhood maltreatment exposure is associated with specific versus broader transdiagnostic increases in disease risk is unknown. Furthermore, it is unclear whether these associations are driven by a smaller subset of children at high risk of a broad range of mental and physical health outcomes, or by many unique individuals with one health outcome. Another knowledge gap exists regarding potential differences in offspring outcomes in association with different forms of maternal childhood maltreatment. Previous research suggests that abuse or threat versus neglect or deprivation might be associated with distinct phenotypes in the exposed individual and the offspring; however, the existing research has not provided consistent results.^11,12^ Although dimensional models of early experience can in principle generate more specific hypotheses about which phenotypes might be associated with certain core dimensions of experiences, such as threat versus deprivation, the most widely available measures assessing childhood maltreatment exposure do not provide sufficient detail about the experience to apply this model.^13^ Furthermore, due to the frequent co-occurrence of maltreatment experiences and the overlap of threat and deprivation characteristics in the commonly used childhood maltreatment categories, it is difficult to disentangle the differential associations of subtypes of childhood maltreatment.^14^ Thus, in this study of a large, racially and ethnically diverse sample combining multiple cohorts across the USA, we aimed to investigate: 1) the degree to which children of mothers with a history of childhood maltreatment exposure had a higher risk of physical health, mental health, or neurodevelopmental problems during childhood and adolescence; 2) whether these children differ in their pattern of comorbid physical health, mental health, and neurodevelopmental problems compared with children of mothers without childhood maltreatment exposure; and 3) whether different maltreatment exposure patterns are differentially associated with child health problems. Asthma, obesity, and allergy were investigated as physical health outcomes of interest, and internalising problems, ADHD, and autism spectrum disorder diagnoses were investigated as mental and neurodevelopmental outcomes of interest. These conditions were selected due to their high prevalence, chronicity, disease burden, and significance for public health.^15,16^

The prevalence and symptomatology of several of these conditions, and their comorbidities, differ depending on sex.^17,18^ Furthermore, evidence suggests sex-specific programming of disease susceptibility.^19^ Therefore, the present study evaluated the moderation of the associations of maternal childhood maltreatment exposure by child sex for all outcomes.

## Methods

### Study design and population

We conducted a retrospective cohort study using data from the Environmental influences on Child Health Outcomes (ECHO) Program,^20^ which was launched in 2016 to investigate the influence of early life exposures on child health and development in 69 cohorts across the USA. Of the 69 cohorts included in the ECHO Program, 21 contributed data for eligible mother–child dyads, with data collection periods initiated between 1999 and 2016. Eligible dyads were those with available data on maternal childhood maltreatment exposure and at least one of the child health outcome measures (described in detail below) in childhood and adolescence. An overview of key characteristics of the included cohorts is provided in the appendix (pp 3–6). For mothers with multiple children in the ECHO dataset, one child was selected at random, irrespective of diagnostic status. There were no specific inclusion or exclusion criteria regarding maternal childhood maltreatment exposure. The study protocol was approved by the local (or central ECHO) institutional review board. Written informed consent or parent’s or guardian’s permission was obtained along with child assent as appropriate for participation in the ECHO-wide Cohort Data Collection Protocol and specific cohorts.

### Exposure measures

Exposure to childhood maltreatment until age 18 years among mothers was characterised using responses to abuse-related and neglect-related items from the Adverse Childhood Experiences questionnaire (first published by Felitti and colleagues^21^) and the Life Stressor Checklist^22^ questionnaire, obtained during prenatal or postnatal visits. Responses were dichotomously coded (yes or no) and further categorised into the following individual types of abuse and neglect: physical abuse, physical neglect, emotional abuse, emotional neglect, and sexual abuse. Responses to the Life Stressor Checklist and Adverse Childhood Experiences questionnaires were mapped onto these types of abuse and neglect and harmonised based on their overlapping constructs. Item descriptions from the Adverse Childhood Experiences and Life Stressor Checklist forms included in the analyses and the correspondingly mapped maternal childhood maltreatment subtypes are included in the appendix (pp 6–7). Two cohorts provided slightly modified versions of the adverse childhood experiences questionnaire, which were also harmonised and mapped onto each of these types of abuse and neglect (appendix pp 1, 6–7).

Individual exposure types were combined into an overall indicator of childhood maltreatment experience (yes or no). Participants were classified as having been exposed to childhood maltreatment if they reported exposure to at least one category of abuse or neglect (irrespective of missing data on other exposure categories), and they were classified as not exposed to childhood maltreatment if they reported no exposure to all categories of abuse and neglect. 383 participants could not be classified into either category of the overall indicator variable due to partial information on the abuse and neglect subtypes; these participants were excluded from the main analyses. The total number of participants with available data and prevalence by each childhood maltreatment subtype are shown in the appendix (p 8).

### Outcome measures

Child health outcome measures comprised autism spectrum disorder, ADHD, obesity, allergy, and asthma diagnoses. Asthma diagnosis in children was captured via parent or caregiver report or self-report of asthma illness or provider diagnosis. Allergy was similarly captured and included any parent or caregiver report or self-report of allergic rhinitis, eczema, or food allergy. Autism spectrum disorder and ADHD diagnoses were captured via parent or caregiver report of diagnosis. Child BMI was harmonised across several instruments with different sources, including researcher-measured height and weight, medical record abstraction, and maternal report. Children with a BMI higher than the age-specific and sex-specific 95th percentile were categorised as obese. Child internalising symptoms were assessed with the preschool-age (1·5–5 years) and school-age (6–18 years) versions of the Child Behavior Checklist.^23,24^ A T-score of 64 or higher was considered indicative of clinically relevant internalising symptoms. For all outcomes, we extracted any available data for children of any age up until 18 years. 502 (12%) children included in the analyses had available data on one disease outcome, 465 (11%) on two disease outcomes, 904 (21%) on three disease outcomes, 208 (5%) on four disease outcomes, 1660 (37%) on five disease outcomes, and 598 (14%) on all six disease outcomes (appendix pp 3–6, 8–9).

### Covariates

Covariates were selected on the basis of known associations with the childhood health outcomes of interest and included maternal-level, paternal-level, and child-level data collected at prenatal, delivery, and postnatal assessments via maternal report. Covariates included child sex (male or female), child race (Black, White, multiracial, or other), child ethnicity (Hispanic or non-Hispanic), maternal and paternal age, maternal education (less than high school, high school, or greater than high school), and combined annual household income (<US$30 000, $30 000–49 999, $50 000–74 999, or ≥$75 000), maternal depression, asthma, ADHD, allergy, autism spectrum disorder diagnosis, and maternal obesity. Race and ethnicity are regarded as social constructs of racial identity that serve to reflect variability in social exposures, including inequities in health and access to health care.^25^ As a result of harmonising data across numerous cohorts, missingness was substantial for some combined variables (range 1·3–42·6%). However, to minimise bias, missing data for all covariates (child age, child race, child ethnicity, maternal age, paternal age, maternal education, and household income) were estimated using multiple imputation.^26^ Estimates were based on the distribution of individual non-missing covariate data using fully conditional specification to account for the combination of continuous and categorical variables; model results were pooled across a series of 25 replications.

### Statistical analysis

The associations between maternal childhood maltreatment and childhood health outcomes were measured using a series of mixed-effect logistic regression models. For each outcome, logistic regression analyses were performed in a subset of the overall sample with available data on the respective diagnosis. The first model included only the unadjusted effect of the exposure (childhood maltreatment) on child health outcome. The second model represents the primary aim of the study (ie, to investigate the degree to which children of mothers with a history of childhood maltreatment exposure had a higher risk of physical health, mental health, or neurodevelopmental problems during childhood and adolescence) and included adjustment for sociodemographic covariates (child age, child race, child ethnicity, maternal age, paternal age, maternal education, and household income). To test whether the association between childhood maltreatment and childhood outcomes differed by child sex, a model was fit that included the interaction of child sex and childhood maltreatment (model 3). To account for potential genetic confounding, all adjusted models were repeated including adjustment for maternal diagnosis of the relevant outcome (models 4 and 5) as a type of sensitivity analysis. Maternal diagnosis of autism spectrum disorder did not show any variation in our sample (ie, none of the mothers reported a diagnosis of autism spectrum disorder), thus sensitivity analyses were not possible for this outcome. A random effect for cohort was included in all models to account for covarying observations within a site. Covariate-adjusted models used imputed covariate data, and results were pooled across 25 distinct imputations.

To characterise patterns in the comorbidity of child health outcomes, we did a latent class analysis with each health outcome included as a binary indicator of latent class membership and the association of maternal childhood maltreatment with latent class membership was explored. Similarly, we did a latent class analysis to characterise patterns of co-occurrence of maternal childhood maltreatment subtypes, with maltreatment types serving as binary indicators of latent class membership, and their associations with child health outcomes were explored (appendix pp 1–2).

All analyses were done using SAS Enterprise Guide (version 7.13) or Mplus (version 8.8); for all model results, adjusted odds ratios (ORs), p values, and 95% CIs are reported. ORs or χ^2^, 95% CIs, and p values for all adjusted and unadjusted associations are presented in tables or the appendix. Interpretation of associations are based on an arbitrary threshold of p≤0·01. The Benjamini and Hochberg method^27^ was used to control the false discovery rate for the primary analyses.

### Role of the funding source

The funder of the study had no role in the study design, data collection, data analysis, data interpretation, or writing of the report.

## Results

A total of 4337 mother–child dyads were included, with an analysis period from November, 2021, to November, 2022. Among the 3954 mothers with full information in the study, 1742 (44%) had experienced exposure to abuse or neglect during their childhood. Sociodemographic characteristics of the overall sample and stratified by childhood maltreatment exposure are shown in table 1. Women with childhood maltreatment exposure were younger at delivery, reported lower education and lower annual household incomes, had a higher proportion of children who were Black or African American or multiracial, and lower proportion of children of Hispanic ethnicity than women without childhood maltreatment exposure. Women with and without childhood maltreatment did not differ by child sex or the age of the biological father at delivery.

The full model including all covariates for child health outcomes is included in the appendix (pp 10–19). In the primary, covariate-adjusted models (model 2), overall group differences were observed for all child health outcomes, with the exception of obesity and allergy (table 2). Specifically, mothers who experienced childhood maltreatment were more likely to have children with internalising problems in the clinical range (OR 2·70 [95% CI 1·95–3·72], p<0·0001), autism spectrum disorder (1·70 [1·13–2·55], p=0·01), ADHD (2·09 [1·63–2·67], p<0·0001), and asthma (1·54 [1·34–1·77], p<0·0001). In model 3, maternal childhood maltreatment was also associated with an increased risk of child obesity in female offspring only (1·69 [1·17–2·44], p=0·005). The association between maternal childhood maltreatment and the other five outcomes did not differ substantially by child sex (model 3). In sensitivity analyses that additionally controlled for maternal diagnosis of the respective child health problem (models 4 and 5), the associations remained similar. Using the Benjamini and Hochberg method^27^ to control the false discovery rate at 0·05 across the 12 tested hypotheses (six main effects, six interaction terms) yielded a critical value of 0·02, below which the null hypothesis can be rejected.

**Figure 1:**
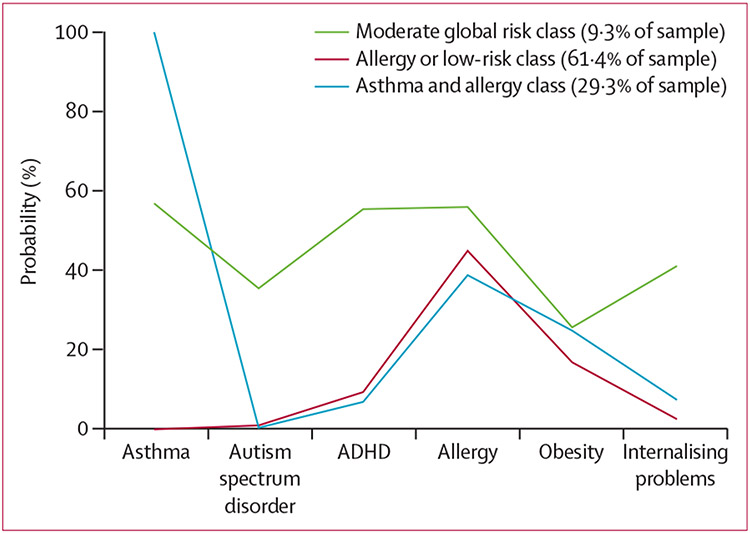
Latent class of child health outcomes ADHD=attention-deficit hyperactivity disorder.

Fit statistics for the latent class model of child health outcomes indicated that a three-class model provided the best fit (appendix p 20). The largest class (61·4% of the sample), referred to as the allergy or low-risk class, was characterised by a low endorsement probability across most of the child health outcome indicators, with the exception of allergy diagnosis. The next largest class (29·3%), referred to as the asthma and allergy class, was characterised by a high probability of an asthma diagnosis, with moderate probabilities of an allergy diagnosis. The third class (9·3%), referred to as the moderate global risk class, was characterised by moderate levels of endorsement across all indicators, including having the highest probability across all classes for autism spectrum disorder diagnosis, ADHD diagnosis, and internalising problems. Figure 1 shows latent class proportions and probabilities of each indicator by class. After the class enumeration process was complete, predictors of class membership were included in the model. Controlling for sex, race, ethnicity, maternal and paternal age, and maternal education and income, maternal childhood maltreatment predicted class membership (table 3; appendix pp 20–21). Specifically, children with mothers who experienced childhood maltreatment were more likely to be in the moderate global risk class than the allergy or low-risk class (OR 3·94 [95% CI 2·26–6·85], p<0·001; table 3). Children with mothers who experienced childhood maltreatment were more likely to be in the moderate global risk class than the asthma and allergy class (3·77 [2·03–7·03], p<0·001). Mothers of children in the moderate global risk class (70·1%) were almost twice as likely to have experienced childhood maltreatment than those with children in the allergy or low-risk class (36·9%) and the asthma and allergy class (38·4%).

**Figure 2:**
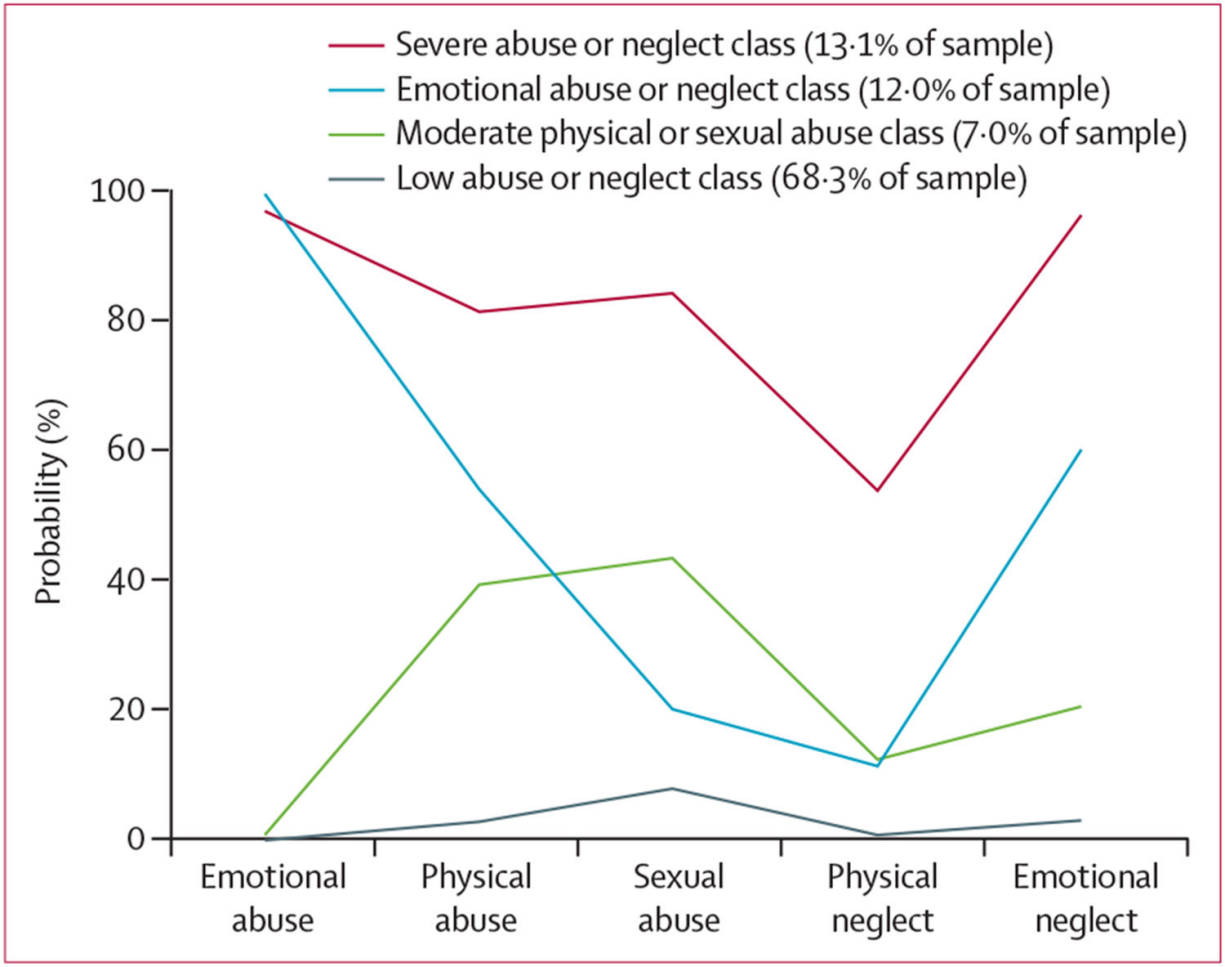
Latent class of maternal maltreatment subtypes

Fit statistics for the latent class model of childhood maltreatment subtypes indicated that a four-class model fit the data best (appendix p 20). The largest class (68·3% of the sample) was characterised by a low probability of endorsement across all forms of childhood maltreatment and was referred to as the low abuse or neglect class. The next largest class (13·1%), referred to as the severe abuse or neglect class, was characterised by a high probability of endorsement across all subtypes. The next largest class (12·0%), referred to as emotional abuse or neglect, is characterised by high probabilities of reported emotional abuse and neglect, but also moderate levels of endorsement for physical abuse. The smallest class (7·0%) is characterised by moderate probabilities of physical and sexual abuse and low probabilities for the other subtypes and is referred to as the moderate physical or sexual abuse class (figure 2). Predictors of childhood maltreatment subtype class membership were differentially associated with child health outcomes. Compared with all other classes, the severe abuse or neglect class was associated with the highest risk for internalising problems, asthma, obesity, and the lowest risk of allergy. Compared with the low abuse or neglect reference class, the emotional abuse or neglect class was associated with higher risk of internalising problems, asthma, obesity, ADHD, and allergy and the moderate physical or sexual abuse class was associated with higher ADHD risk. Table 4 shows the group comparisons between classes regarding their associations with each child health outcome.

## Discussion

Maltreatment in childhood has been associated with poor health outcomes across the life course. However, the hypothesis that childhood-maltreatment-related increases in disease risk might extend beyond the individual exposed is less widely acknowledged. Specific examples of intergenerational associations of adversity have been largely confined to analyses of highly specific exposures—eg, among Holocaust survivors^28^ or outcomes representing unique aspects of mental or physical health.^29-31^ The findings of this study add to and extend this research by demonstrating that reported childhood maltreatment exposure in mothers was associated with a 54–170% increased risk for several physical health, mental health, and neurodevelopmental problems in offspring throughout childhood and adolescence across a large, combined sample of multiple US cohorts. The risk increases in association with maternal childhood maltreatment extended to health outcomes with divergent biological origins and phenotypes, such as asthma and autism spectrum disorder. Furthermore, maternal childhood maltreatment was associated with a morbidity pattern characterised by a broad, transdiagnostic risk across all child health outcomes examined, suggesting that several health problems cluster in some individuals rather than different individuals with distinct health problems. For most health outcomes, a dose–response relationship was identified with maternal childhood maltreatment, whereby childhood maltreatment subtype patterns characterised by multiple exposures (severe abuse or neglect and emotional abuse or neglect) were generally associated with the largest increases in risk. The majority of associations between maternal childhood maltreatment and offspring health did not seem to be sex-specific, with the exception of obesity, which was more likely to occur only in female offspring of mothers exposed to maltreatment in childhood.

Although the observed increase in disease risk among children of mothers reporting childhood maltreatment exposure is substantial, it is important to note that many children of mothers who reported exposure to childhood maltreatment did not have these health problems and potentially were able to avert the intergenerational transmission of risk associated with childhood maltreatment. Further research into determinants of such resilience might be an important basis for designing interventions for children at risk of intergenerational transmission of risk.

Although the observational nature of our study does not permit causal inference between maternal childhood maltreatment exposure and child health outcomes, it was intended to investigate the nature and strength of the association of variation in reported maternal childhood maltreatment experiences with subsequent child mental, physical, and neurodevelopmental outcomes to provide the best possible evidence that either supports or refutes the underlying hypothesis.

The results are mostly consistent with previous studies that have reported associations of maternal childhood maltreatment with singular child mental and physical health outcomes in terms of the direction of the association and the effect size,^29,30,32^ adding confidence to previously demonstrated associations with this diverse cross-cohort sample. Contrary to the findings of a previous study,^32^ maternal reported childhood maltreatment exposure was not associated with an overall increased risk for allergy in the present study. Instead, the association between maternal childhood maltreatment and allergy was dependent on the specific pattern of exposure the mother reported. The severe abuse or neglect class, which was characterised by the highest probabilities for all childhood maltreatment subtypes, was associated with lower risk for allergy compared with all other classes, whereas the emotional abuse or neglect class was associated with a higher risk of allergy. The mechanism underlying these differences in directionality is unclear but might be related to the non-specific nature of the definition of allergy (including several forms of atopic disease, which might be differentially associated with childhood maltreatment exposure patterns). The prevalence of allergy was relatively high in the present study (43%), which might also be associated with the broad definition of allergy used, or over-reporting of milder forms of allergic diseases. Furthermore, allergy often occurs concurrently with asthma,^33^ which is supported by the presence of the asthma and allergy class in the latent class analysis. Similarly, the prevalence of asthma was high (46%), which can partly be explained by the inclusion of a cohort that specifically recruited children with a familial history of asthma.

Compared with other health outcomes, the association between autism spectrum disorder and maternal childhood maltreatment exposure overall was relatively weaker and was not strongly associated with a specific childhood maltreatment exposure pattern. This finding suggests that the contribution of genetic factors and other environmental factors that are not related to maternal childhood maltreatment exposure might be stronger for autism spectrum disorder than for the other investigated health problems. Furthermore, the probability of an ADHD diagnosis was not highest among offspring of women reporting the highest number of abuse or neglect categories, but among those reporting exposure to emotional abuse and neglect or physical and sexual abuse, specifically. Future studies should focus on the characterisation of the specific prenatal and postnatal transmission pathways of distinct patterns of maternal childhood maltreatment exposure.

Our finding that the association of maternal childhood maltreatment with obesity was only observed in female offspring is novel. Previous studies reporting an association between maternal childhood maltreatment and child obesity did not investigate moderation of this association by child sex,^31,34^ which precludes a direct comparison of the respective findings; however, a systematic review on the association between childhood maltreatment and obesity in exposed individuals suggests that women might be more susceptible to the effect of childhood maltreatment on obesity than men,^35^ and our findings suggest this sex-specificity might extend to intergenerational effects. Mechanisms of sex-specific programming of disease susceptibility might involve sex hormones (eg, estradiol stimulates the activity of the hypothalamic–pituitary–adrenal axis),^36^ placental mechanisms (sexual dimorphism exists regarding placental growth, structure, and gene and non-coding RNA expression),^37^ or extrinsic factors related to sex or gender (eg, gender-specifc parenting attitudes, behaviours, and modelling).

Exposure to childhood maltreatment (ie, any type of abuse or neglect) was reported by around 44% of participants in our sample, which is towards the upper end of previously reported prevalence ranges.^1,2,38^ This relatively high proportion might be partly explained by the procedure used to compute the childhood maltreatment variable, which categorised participants into the exposure group if they endorsed exposure to any type of abuse or neglect, irrespective of missing information on other categories. Participants who did not have complete data for all abuse and neglect categories were excluded from the non-exposure group to ensure the absence of any type of abuse and neglect in this group. Another possibility might be related to the population characteristics of the included cohorts, which cover diverse regions and populations from a variety of racial and ethnic backgrounds across the USA. Although none of the cohorts specifically selected women with a history of childhood maltreatment, compared with the general population of the USA, the study sample included more individuals from minoritised racial and ethnic groups and with lower reported income. Since childhood maltreatment is associated with multiple forms of socioeconomic disadvantage,^39^ these characteristics might partly explain the higher prevalence of childhood maltreatment in this sample. Although we consider the inclusion of a large proportion of individuals from population groups who are frequently underrepresented in scientific studies an advantage of our sample, these characteristics and the enrichment of some cohorts for individuals with specific outcomes (eg, individuals born preterm or those with familial asthma; appendix pp 2–6) have implications for generalisability and thus, the present findings should be extrapolated with caution.

It was beyond the scope of the present study to discern specific mechanisms and pathways of intergenerational transmission. Multiple mechanisms are likely to be involved considering the disparate health outcomes affected, but shared risk pathways might also exist. One plausible mechanism that has gained considerable attention is epigenetic inheritance (ie, germline transmission of epigenetic information between generations), although conclusive evidence for the existence of epigenetic inheritance in humans is absent. Additionally, evidence suggests that the biological correlates of childhood maltreatment might be carried forward into pregnancy and affect maternal antepartum health and gestational biology (including maternal stress physiology, immune function, and cardiometabolic health), with implications for child health outcomes,^7,8^ possibly through a fetal programming mechanism.^40,41^ The quality of the child’s postnatal environment is another important potential mediator of intergenerational transmission—eg, psychosocial challenges related to histories of maltreatment, such as maternal psychopathology and difficulty providing high-quality parenting as a consequence of childhood-maltreatment-related personal, social, and socioeconomic constraints, could affect child health outcomes.^42,43^

The study is not without limitations. Child health outcomes were assessed at different ages across childhood and adolescence. Although all investigated health outcomes are characterised by a high level of chronicity, conditions can be transient and might differ in terms of age of onset; therefore, diagnostic status might fluctuate across childhood and adolescence.^44,45^ Thus, we cannot preclude that some diagnoses were missed because they had not yet manifested at the time of assessment or because a transient condition was no longer present and not reported retrospectively.

Additionally, child health outcomes were based mainly (albeit not exclusively) on maternal or caregiver report. Maternal or caregiver report of child health problems is frequently used in studies investigating prenatal and postnatal psychosocial influences on child health and development, and almost as frequently the concern is raised that responses might be biased as a result of the caregiver’s own current psychopathology. However, a 2021 study empirically tested these potential biases in a large sample and found only minimal evidence for biases in maternal reports of child psychopathology.^46^ Nonetheless, it remains possible that other childhood-maltreatment-related maternal states and behaviours could have influenced maternal reporting.

Furthermore, the relevance of paternal childhood maltreatment experiences on child health has been neglected in previous research. In our study, associations between paternal exposures of childhood maltreatment and child health could not be investigated due to low availability of data on paternal experiences. Future research should investigate the combined and independent intergenerational effects of maternal and paternal childhood maltreatment exposure.

The possibility of a genetic confound that could predispose related family members for mental and physical health and neurodevelopmental problems and at the same time increase the probability of maltreatment exposure should not be ruled out.^47,48^ We attempted to address this possibility of a genetic confound in sensitivity analyses by adjusting the models for maternal diagnosis of the respective health outcome (with the exception of autism spectrum disorder, for which no maternal diagnosis was reported). Although maternal diagnosis was a strong predictor of child diagnosis for most health problems (except allergy), the associations between maternal childhood maltreatment and child health remained essentially unchanged.

The findings of this study provide empirical support for a broad intergenerational impact of maternal childhood maltreatment exposure on multiple health outcomes within individuals. Two systematic reviews and meta-analyses suggest that childhood adversity, including childhood maltreatment, is a major contributing factor to mortality and morbidity in the USA and Europe, leading to an enormous financial burden corresponding to costs between 1% and 6% of a country’s gross domestic product.^6,49^ Intergenerational transmission of the adverse health outcomes associated with childhood maltreatment multiplies the number of affected individuals, thus further exacerbating the health and financial costs associated with childhood maltreatment.

The broad relevance of childhood maltreatment for health across generations underscores the value and benefit of preventing childhood maltreatment and mitigating its impact. Furthermore, screening for maternal childhood maltreatment experiences as part of trauma-informed prenatal care might help to identify vulnerable populations during a unique window of opportunity for interventions promoting both maternal and child health. However, in addition to assessing past challenges, screening should also focus on an individual’s strengths to identify resources supporting resilience that could counteract the intergenerational transmission of health risk.

## Supplementary Material

1

## Figures and Tables

**Table 1:** Characteristics of the study sample

	Study population (n=4337)	Exposed to childhood maltreatment (n=1742)	Not exposed to childhood maltreatment (n=2212)	p value
Number of pregnancies	4337	1742	2212	··
Maternal age at delivery, years				
Participants with available data	4311 (99·4%)	1733 (99·5%)	2203 (99·6%)	··
Mean (SD)	28·74 (6·15)	28·31 (6·21)	29·53 (5·85)	<0·0001
Paternal age at delivery, years				
Participants with available data	2921 (67·4%)	1154 (66·2%)	1626 (73·5%)	··
Mean (SD)	31·72 (7·09)	31·54 (7·15)	31·54 (7·15)	0·1
Race of child				
Participants with available data	4112 (94·8%)	1682 (96·6%)	2107 (95·3%)	··
White	2156/4112 (52·4%)	880/1682 (52·3%)	1234/2107 (58·6%)	··
Black or African American	1201/4112 (29·2%)	496/1682 (29·5%)	463/2107 (22·0%)	··
Multiracial	519/4112 (12·6%)	226/1682 (13·4%)	241/2107 (11·4%)	··
Other	236/4112 (5·7%)	80/1682 (4·8%)	169/2107 (8·0%)	<0·0001
Ethnicity of child				
Participants with available data	4282 (98·7%)	1726 (99·1%)	2184 (98·7%)	
Hispanic	1173/4282 (274%)	445/1726 (25·8%)	655/2184 (30·0%)	··
Non-Hispanic	3109/4282 (72·6%)	1281/1726 (74·2%)	1529/2184 (70·0%)	0·004
Maternal education				
Participants with available data	2767 (63·8%)	1111 (63·8%)	1564 (70·7%)	··
Less than high school	256/2767 (9·3%)	113/1111 (10·2%)	136/1564 (8·7%)	··
High school degree, GED, or equivalent	438/2767 (15·8%)	201/1111 (18·1%)	216/1564 (13·8%)	··
Some college education (no degree), or higher	2073/2767 (74·9%)	797/1111 (71·7%)	1212/1564 (77·5%)	0·003
Combined household income (US$) within 1 year of birth				
Participants with available data	3078 (71·0%)	1271 (73·0%)	1460 (66·0%)	··
<30 000	970/3078 (31·5%)	422/1271 (33·2%)	333/1460 (22·8%)	··
30 000–49 999	534/3078 (17·3%)	266/1271 (20·9%)	188/1460 (12·9%)	··
50 000–74 999	419/3078 (13·6%)	181/1271 (14·2%)	217/1460 (14·9%)	··
≥75 000	1155/3078 (37·5%)	402/1271 (31·6%)	722/1460 (49·5%)	<0·0001
Sex of child				
Participants with available data	4328 (99·8%)	1740 (99·9%)	2205 (99·7%)	··
Male	2237/4328 (51·7%)	876/1740 (50·3%)	1133/2205 (51·4%)	··
Female	2091/4328 (48·3%)	864/1740 (49·7%)	1072/2205 (48·6%)	0·52

Data are n (%) or mean (SD). Childhood maltreatment was defined as exposure to abuse or neglect. 383 participants from the total population could not be classified as having been exposed to childhood maltreatment or not exposed to childhood maltreatment, thus frequencies presented for the two exposure groups do not sum to the frequencies of the total study population. GED=General Educational Development.

**Table 2:** Mixed-effect logistic regression model analysis of effects of childhood maltreatment and the interaction by child sex and child outcome

	Model 1 (unadjusted model)		Model 2 (adjusted model)		Model 3 (childhood maltreatment and child sex interaction)		Model 4 (maternal diagnosis outcome)		Model 5 (maternal diagnosis and child sex interaction)	
					
	OR (95% CI)	p value	OR (95% CI)	p value	OR (95% CI)	p value	OR (95% CI)	p value	OR (95% CI)	p value
**Internalising problems (n=2298)**										
Childhood maltreatment	4·04 (2–56–6·38)	<0·0001	2·70 (1·95–3·72)	<0·0001	2·37 (1·54–3·64)	<0·0001	2·29 (1·64–3·21)	<0·0001	2·01 (1·29–3·12)	0·002
Childhood maltreatment × female sex interaction	··	··	··	··	1·33 (0·70–2·52)	0·38	··	··	1·34 (0·70–2·54)	0·37
**Asthma (n=4074)**										
Childhood maltreatment	1·29 (1·06–1·58)	0·01	1·54 (1·34–1·77)	<0·0001	1·52 (1·26–1·84)	<0·0001	1·46 (1·27–1·67)	<0·0001	1·46 (1·21–1·77)	<0·0001
Childhood maltreatment × female sex interaction	··	··	··	··	1·03 (0·78–1·35)	0·84	··	··	0·99 (0·76–1·30)	0·96
**Obesity (n=3325)**										
Childhood maltreatment	1·14 (0·89–1·45)	0·31	1·11 (0·92–1·34)	0·27	0·87 (0·68–1·12)	0·29	1·12 (0·93–1·35)	0·24	0·87 (0·67–1·11)	0·26
Childhood maltreatment × female sex interaction	··	··	··	··	1·69 (1·17–2·44)	0·005	··	··	1·75 (1·21–2·53)	0·003
**Autism spectrum disorder (n=2362)**										
Childhood maltreatment	1·50 (0·88–2·55)	0·14	1·70 (1·13–2·55)	0·01	1·89 (1·17–3·04)	0·009	··	··	··	··
Childhood maltreatment × female sex interaction	··	··	··	··	0·66 (0·26–1·66)	0·38	··	··	··	··
**ADHD (n=2386)**										
Childhood maltreatment	2·31 (1·70–3·15)	<0·0001	2·09 (1·63–2·67)	<0·0001	2·01 (1·49–2·70)	<0·0001	2·07 (1·61–2·65)	<0·0001	2·00 (1·49–2·70)	<0·0001
Childhood maltreatment × female sex interaction	··	··	··	··	1·13 (0·67–1·92)	0·64	··	··	1·11 (0·65–1·87)	0·71
**Allergy (n=1947)**										
Childhood maltreatment	1·11 (0·85–1·46)	0·44	0·87 (0·72–1·05)	0·15	0·86 (0·66–1·11)	0·25	0·78 (0·63–0·95)	0·02	0·80 (0·60–1·06)	0·11
Childhood maltreatment × female sex interaction	··	··	··	··	1·03 (0·72–1·49)	0·87	··	··	0·94 (0·64–1·40)	0·77

OR=odds ratio. ADHD=attention-deficit hyperactivity disorder.

**Table 3:** Associations between maternal childhood maltreatment and child health outcome latent classes

	Moderate global risk class *vs* asthma and allergy class		Allergy or low-risk class *vs* asthma and allergy class		Moderate global risk class *vs* allergy or low-risk class	
	OR (95% CI)	p value	OR (95% CI)	p value	OR (95% CI)	p value
Unadjusted model	2·91 (1·71–4·96)	<0·001	0·69 (0·59–0·81)	<0·001	4·21 (2·57–6·92)	<0·001
Adjusted model	3·77 (2·03–7·03)	<0·001	0·96 (0·77–1·19)	0·70	3·94 (2·26–6·85)	<0·001

61·4% of the sample were referred to as the allergy or low-risk class, 29·3% of the sample were referred to as the asthma and allergy class, and 9·3% of the sample were referred to as the moderate global risk class. The adjusted model controlled for maternal age, paternal age, race or ethnicity, sex, maternal education, and income. OR=odds ratio.

**Table 4:** Child health outcome probabilities for each latent maternal childhood maltreatment subtype class and comparisons between the latent classes for each child health outcome

	Probability of class				Comparison between classes						
	Severe abuse or neglect class	Emotional abuse or neglect class	Moderate physical or sexual abuse class	Low abuse or neglect class	Overall	Severe abuse or neglect class *vs* emotional abuse or neglect class	Severe abuse or neglect class *vs* moderate physical or sexual abuse class	Severe abuse or neglect class *vs* low abuse or neglect class	Emotional abuse or neglect class *vs* moderate physical or sexual abuse class	Emotional abuse or neglect class *vs* low abuse or neglect class	Moderate physical or sexual abuse class *vs* low abuse or neglect class
Internalising problems	0·257	0·114	0·115	0·054	29·61, p<0·001	4·13, p=0·042	4·01, p=0·045	11·66, p=0·001	0·00, p=0·972	7·20, p=0·007	3·14, p=0·076
Asthma	0·858	0·273	0·464	0·43	134·59, p<0·001	122·26, p<0·001	42·50, p<0·001	119·35, p<0·001	16·08, p<0·001	27·87, p<0·001	0·55, p=0·460
Obesity	0·347	0·126	0·232	0·197	25·64, p<0·001	22·55, p<0·001	4·72, p<0·001	22·12, p<0·001	5·57, p=0·018	6·80, p=0·009	0·61, p=0·437
Autism spectrum disorder	0·144	0·046	0·046	0·042	6·33, p=0·097	3·49, p=0·062	3·55, p=0·060	5·17, p=0·023	0·00, p=0·989	0·07, p=0·796	0·04, p=0·847
ADHD	0·096	0·246	0·212	0·096	32·90, p<0·001	3·73, p=0·053	2·22, p=0·136	0·00, p=0·995	0·59, p=0·442	26·67, p<0·001	7·50, p=0·006
Allergy	0·305	0·657	0·515	0·414	24·60, p<0·001	23·59, p<0·001	8·69, p=0·003	9·06, p=0·003	3·50, p=0·061	19·31, p<0·001	2·39, p=0·123

Data are probability of a child health outcome within each latent class, or χ^2^, p value for comparison. 68·3% of the sample was characterised as the low abuse or neglect class, 13·1% were characterised as the severe abuse or neglect class, 12·0% were characterised as the emotional abuse or neglect class, and 7·0% were characterised as the moderate physical or sexual abuse class. ADHD=attention-deficit hyperactivity disorder.

## Data Availability

De-identified data from the Environmental influences on Child Health Outcomes (ECHO) Program are available through the Data and Specimen Hub (DASH) of the Eunice Kennedy Shriver National Institute of Child Health and Human Development. Researchers can request access to these data by creating a DASH account and submitting a data request form.

## Research in context

### Evidence before this study

Adverse experiences during a parent’s own childhood might affect their offspring’s development and health. We searched PubMed from database inception until March 16, 2022 for published articles using the search terms “(intergenerational transmission) AND ((maternal) OR (offspring)) AND ((childhood maltreatment) OR (childhood abuse) OR (early adversity) OR (adverse childhood experiences) OR (childhood trauma)) AND ((psychopathology) OR (physical health) OR (obesity) OR (BMI) OR (asthma) OR (allergy) OR (atopy) OR (internalizing) OR (depression) OR (externalizing) OR (adhd) OR (autism) OR (asd))”. We supplemented the search by including further relevant studies from the reference lists of the identified articles. We identified 14 studies investigating the association between maternal experiences of childhood maltreatment and child physical and mental health outcomes. Evidence from these studies indicates an increased risk for a range of physical and mental health problems in children of mothers exposed to childhood maltreatment; however, whether childhood maltreatment is associated with a specific or a broader transdiagnostic risk is unknown. More research is needed to elucidate whether maternal exposure to different patterns of maltreatment subtypes is associated with different health outcomes in offspring.

### Added value of this study

To our knowledge, this is the first study to comprehensively investigate the association between maternal childhood maltreatment and a broad range of physical and mental health conditions and comorbidities in offspring in a large sociodemographically and ethnically diverse sample across the USA. Furthermore, we report the association between distinct patterns of maltreatment exposure and health outcomes in offspring. We observed that across childhood and adolescence, children of mothers who reported a history of childhood maltreatment were more likely to have reported diagnoses of asthma, attention-deficit hyperactivity disorder, and autism spectrum disorder, and clinically relevant symptoms of internalising problems. Child obesity was more prevalent among female, but not male, offspring of women exposed to childhood maltreatment. Additionally, maternal childhood maltreatment increased the likelihood of offspring belonging to a latent class that was characterised by elevated probabilities for all health outcomes. Some specificity was identified in different patterns of childhood maltreatment subtypes with regard to their associations with child health problems; however, a dose-response relationship was also identified, such that the pattern characterised by exposure to multiple forms of maltreatment across all subtypes was associated with the highest risk for most health outcomes.

### Implications of all the available evidence

This study advances our understanding of the relevance of childhood maltreatment as a contributing factor to health across generations. The findings highlight the potential widespread benefits of preventing childhood maltreatment and use of childhood maltreatment screening to directly target interventions to mitigate the intergenerational impact of adversity. Future research to identify mechanistic pathways of transmission will be important for refining potential targets of intervention.
