# Supplementary material for: Intergenerational transmission of the effects of maternal exposure to childhood maltreatment in the USA: a retrospective cohort study
Source: Lancet Public Health. Author manuscript; Available in PMC 2023 Mar 3. (PMC9982823; doi:10.1016/S2468-2667(23)00025-7)
Supplement: 1 [file NIHMS1877532-supplement-1.pdf]

THE LANCET
Public Health

Supplementary appendix

This appendix formed part of the original submission and has been peer reviewed. We post it as supplied by the authors.

Supplement to: Moog NK, Cummings PD, Jackson KL, et al. Intergenerational transmission of the effects of maternal exposure to childhood maltreatment in the USA: a retrospective cohort study. *Lancet Public Health* 2023; **8:** e226–37.

Supplemental Material

Maternal Childhood Maltreatment Measures

For the ACEs questionnaire version, which was available in 14 cohorts ("primary version"), each domain of maternal CM was assessed via two questions. For this ACEs questionnaire version, a valid, non-missing response for both the primary and secondary exposure indicator was required for inclusion. For the two cohorts that used a modified version of the ACEs questionnaire, each exposure domain was captured by one question that combined the primary and secondary exposure indicators (see Table S1). For participants who responded to these modified ACEs questionnaires, a valid, non-missing response to each singular exposure type indicator was sufficient for inclusion.

For the LSC questionnaire that was used in 7 cohorts, mothers were classified as having been exposed to emotional abuse or physical neglect if they endorsed the respective item along with a follow-up question specifying exposure before the age of 18 years. The other abuse and neglect questions included age specifying criteria within the question.

Statistical Analysis

To explore patterns of comorbidity of child health outcomes, a latent class analysis was performed with each health outcome included as a binary indicator of latent class membership. The number of classes was selected using standard fit indices, including the Bayesian information criterion (BIC), the Lo-Mendell-Rubin adjusted likelihood ratio test (LMR-LRT), and substantive interpretation.[39] The association of maternal CM with latent class membership was explored utilizing the manual three-step approach[40,41], which allows for the final model to account for measurement error. Standard errors were adjusted for clustering at the cohort level via sandwich estimators. Missing data on the child health outcome indicators were accounted for using full information maximum likelihood estimation. Current research standards indicate data can be treated as missing at random (MAR) when the missing data are unrelated to the outcome of interest.[1,2] Because of the patterns of missing data in addition to the fact that

there were no demographic differences in our analytic sample among those who have outcome data versus those who do not, we can assume the MAR criterion to be fulfilled.

Similarly, a latent class analysis was performed to characterize patterns of co-occurrence of maternal CM subtypes, with maltreatment types serving as binary indicators of latent class membership. The number of latent classes was determined based on the standard practices described above. Associations between CM subtype class membership and child health outcomes were explored using the single step approach in MPlus.

Table S1. Key characteristics of ECHO cohorts contributing data.

**Cohort Name**	**Data collection year initiation**	**Location of data collection**	**Design**	**Target population**	**Number of children included in analyses**
NICU Hospital Exposure and Long-Term Health	2011	New York	Longitudinal cohort initiated during pregnancy	Birth weight under 1500g or gestational age 28 through 32 weeks, NICU population	Asthma: 25<br>ASD: 16<br>ADHD: 16<br>Allergy: 10<br>Obesity: 15<br>CBCL: 19
Developmental Impact of NICU Exposures (DINE)	2010	California, New York, Ohio, North Carolina, Texas, Indiana, Tennessee, Missouri, South Carolina, Illinois, Florida, Arkansas, Minnesota, Washington	Longitudinal cohort initiated at birth, Clinical trial (randomized double-blinded clinical trial of late surfactant and inhaled nitric oxide (iNO) vs iNO-alone in extremely preterm newborns)	Gestational age 23 through 28 weeks; Clinical trial part of the sample: >/= 28 weeks; Intubated and mechanically ventilated at 7-14 days; Plan to treat with inhaled nitric oxide, NICU population	Asthma: 123<br>ASD: 120<br>ADHD: 119<br>Allergy: 115<br>Obesity: 121<br>CBCL: 33
Family Life Project	2003	North Carolina, Pennsylvania	Longitudinal cohort initiated during pregnancy	Must have given birth in target hospitals, general population	Asthma: 677<br>ASD: 666<br>ADHD: 665<br>Allergy: 16<br>Obesity: 763<br>CBCL: 669

BAMBAM & MINNIE (two cohorts combined)	2010	Rhode Island	Longitudinal cohort initiated when infants were approximately 3 months of age	Healthy children, excluded children born preterm, general population	Asthma: 280<br>ASD: 98<br>ADHD: 119<br>Allergy: 53<br>Obesity: 225<br>CBCL: 192
Boricua Youth Study	2001	New York, Puerto Rico	Longitudinal cohort initiated when children ranged from 5 to 13 years old	Children living in specific geographic areas, general population living in urban areas	Asthma: 52<br>ASD: 13<br>ADHD: 13<br>Allergy: 23<br>Obesity: 9<br>CBCL: 46
PETALS (Pregnancy Environment and Lifestyle Study)	2013	California	Longitudinal cohort initiated during pregnancy	Pregnant women receiving care within a specific medical system, general population	Asthma: 184<br>ASD: 184<br>ADHD: 184<br>Allergy: 64<br>Obesity: 181<br>CBCL: 182
KPRB (Kaiser Permanente Research Bank)	2007	California	Longitudinal cohort initiated during pregnancy	Pregnant women receiving care within a specific medical system, general population	Asthma: 113<br>ASD: 113<br>ADHD: 113<br>Allergy: 39<br>Obesity: 111<br>CBCL: 113
Urban Environment and Childhood Asthma	2004	Maryland, Massachusetts, New York, Missouri	Longitudinal disease specific birth cohort initiated during pregnancy	Pregnant women delivering at specific hospitals with a parental history of asthma, allergic rhinitis (hay fever), or eczema (atopic dermatitis)	Asthma: 536<br>ASD: 0<br>ADHD: 0<br>Allergy: 536<br>Obesity: 531<br>CBCL: 0
MADRES	2016	California	Longitudinal cohort initiated during pregnancy	Pregnant women with singleton births, general population	Asthma: 335<br>ASD: 0<br>ADHD: 0<br>Allergy: 73<br>Obesity: 0<br>CBCL: 93

Pittsburgh Girls Study	2000	Pennsylvania	Longitudinal cohort initiated during maternal childhood	Girls living in Pittsburgh, general population	Asthma: 48<br>ASD: 9<br>ADHD: 9<br>Allergy: <5<br>Obesity: <5<br>CBCL: 0
ECHO-NOVI	2014	Michigan, Hawaii, Missouri, Rhode Island, California, North Carolina	Longitudinal cohort initiated when children were infants	Infants born less than 30 weeks gestational age, NICU population	Asthma: 161<br>ASD: 150<br>ADHD: 150<br>Allergy: 56<br>Obesity: 162<br>CBCL: 172
Vitamin C to Decrease Effects of Smoking in Pregnancy on Infant Lung Function	2013	Indiana, Oregon, Washington	Clinical Trial initiated during pregnancy with longitudinal follow-up	Pregnant women receiving care at specific medical centers, current smokers, general population	Asthma: 189<br>ASD: 184<br>ADHD: 185<br>Allergy: 189<br>Obesity: 180<br>CBCL: 166
In-Utero Smoke, Vitamin C, and Newborn Lung Function	2007	Oregon, Washington	Clinical Trial initiated during pregnancy with longitudinal follow-up	Pregnant women receiving care at specific medical centers, current smokers, general population	Asthma: 92<br>ASD: 87<br>ADHD: 89<br>Allergy: 92<br>Obesity: 89<br>CBCL: 67
University of California Davis – BRSC	2003	California	Longitudinal cohort initiated during pregnancy	HR group: Status as a younger sibling of a child with confirmed ASD; LR group: At least one older sibling with no evidence of ASD or other neurodevelopmental disorder, Autism familial-enriched risk	Asthma: 11<br>ASD: 11<br>ADHD: 11<br>Allergy: 6<br>Obesity: 10<br>CBCL: 7

University of California – MARBLES	2006	California	Longitudinal cohort initiated during pregnancy	Pregnant women with an older biological child diagnosed with ASD, Autism familial-enriched risk	Asthma: 29<br>ASD: 28<br>ADHD: 28<br>Allergy: 15<br>Obesity: 20<br>CBCL: 29
Infant Brain Imaging Study – IBIS	2007	North Carolina, Washington, Pennsylvania	Longitudinal cohort initiated when children were infants	HR group: Status as a younger sibling of a child with confirmed ASD; LR group: At least one older sibling with no evidence of ASD or other neurodevelop mental disorder, Autism familial-enriched risk	Asthma: <5<br>ASD: <5<br>ADHD: <5<br>Allergy: <5<br>Obesity: 0<br>CBCL: 0
Early Autism Risk Longitudinal Investigation – EARLI	2009	Maryland	Longitudinal cohort initiated during pregnancy	Pregnant women with an older biological child diagnosed with ASD, Autism familial-enriched risk	Asthma: 22<br>ASD: 22<br>ADHD: 21<br>Allergy: 11<br>Obesity: 18<br>CBCL: 22
Rochester	2016	New York	Longitudinal cohort initiated during pregnancy	Pregnant women with singleton births, general population	Asthma: 234<br>ASD: 0<br>ADHD: 0<br>Allergy: 232<br>Obesity: 122<br>CBCL: 0
Project Viva	1999	Massachusetts	Longitudinal cohort initiated during pregnancy	Receive prenatal care at one of the selected practice and plan to deliver at one of two study	Asthma: 382<br>ASD: 380<br>ADHD: 382<br>Allergy: 382<br>Obesity: 378<br>CBCL: 0

				hospitals, general population	
Programming of Intergeneration Stress of Mechanisms	2011	Massachusetts, New York	Longitudinal cohort initiated during pregnancy	Pregnant women with singleton births, general population	Asthma: 697<br>ASD: 280<br>ADHD: 281<br>Allergy: 372<br>Obesity: 401<br>CBCL: 488

Table S2: Assessment and harmonization of maternal childhood maltreatment experiences

Construct	Instrument	Item
**Physical abuse**	ACE (primary)	a. Did a parent or other adult in the household often push, grab, slap, or throw something at you?<br>b. Did a parent or other adult in the household ever hit you so hard that you had marks or were injured?
	ACE (modified)	Did a parent or other adult in the household often push, grab, slap, or throw something at you or ever hit you so hard that you had marks or were injured?
	LSC	Before age 18, were you ever abused or physically attacked (not sexually) by someone you knew (for example, a parent, boyfriend, or husband, hit, slapped, choked, burned, or beat you up)?
**Sexual abuse**	ACE (primary)	a. Did an adult or person at least 5 years older than you ever touch or fondle you or have you touch their body in a sexual way?<br>b. Did an adult or person at least 5 years older than you ever attempt or actually have oral, anal, or vaginal intercourse with you?
	ACE (modified)	Did an adult or person at least 5 years older than you ever touch or fondle you or have you touch their body in a sexual way or try to or actually have oral, anal, or vaginal sex with you?
	LSC	a. Before age 18, were you ever touched or made to touch someone else in a sexual way because he/she forced you in some way or threatened to harm you if you didn't?<br>b. Before age 18, did you ever have sex (oral, anal, genital) when you didn't want to because someone forced you in some way or threatened to hurt you if you didn't?

**Emotional abuse**	ACE (primary)	a. Did a parent or other adult in the household often swear at you, insult you, put you down, or humiliate you?<br>b. Did a parent or other adult in the household often act in a way that made you afraid that you might be physically hurt?
	ACE (modified)	Did a parent or other adult in the household often swear at you, insult you, put you down, or humiliate you or act in a way that made you afraid that you might be physically hurt?
	LSC	Have you ever been emotionally abused or neglected (for example, being frequently shamed, embarrassed, ignored, or repeatedly told that you were 'no good')?
**Physical neglect**	ACE (primary)	a. Did you often feel that you didn't have enough to eat, had to wear dirty clothes, and had no one to protect you?<br>b. Did you often feel that your parents were too drunk or high to take care of you or take you to the doctor if you needed it?
	ACE (modified)	Did you often feel that you didn't have enough to eat, had to wear dirty clothes, and had no one to protect you or that your parents were too drunk or high to take care of you or take you to the doctor if you needed it?
	LSC	Have you ever been physically neglected (for example, not fed, not properly clothed, or left to take care of yourself when you were too young or ill)?
**Emotional neglect**	ACE (primary)	a. Did you often feel that no one in your family loved you or thought you were important or special?<br>b. Did you often feel that your family didn't look out for each other, feel close to each other, or support each other?
	ACE (modified)	Did you often feel that no one in your family loved you or thought you were important or special or that your family didn't look out for each other, feel close to each other, or support each other?
	LSC	n/a

Table S3: Data availability and prevalence of maternal childhood maltreatment exposure categories

Childhood maltreatment	N (%) with data	N (%) reporting exposure
Emotional Abuse (y/n)	3754 (86·56%)	841 (22·40%)
Physical Abuse (y/n)	4309 (99·35%)	964 (22·37%)
Sexual Abuse (y/n)	3752 (86·51%)	765 (20·39%)
Physical Neglect (y/n)	3752 (86·51%)	307 (8·18%)
Emotional Neglect (y/n)	3768 (86·88%)	807 (21·42%)
Any Abuse (y/n)	3973 (91·61%)	1603 (40·35%)
Any Neglect (y/n)	3737 (86·17%)	887 (23·74%)
Any Abuse or Neglect (CM, y/n)	3954 (91·17%)	1742 (44·06%)

Note. CM, childhood maltreatment.

Table S4: Child health outcomes and age at diagnosis

Outcome (measure detail)	N (%) with data	N (%) reporting dx	Mean (SD) age among those with dx	Age range (Min, Max)
Internalizing problems (age at testing)	2298 (52·99%)	204 (8·88%)	8·87 (5·04)	(2, 17)
Asthma (age at dx)	4047 (93·31%)	1844 (45·26%)	4·34 (4·88)	(0·03, 17·66)
Obesity (age at testing)	3325 (76·67%)	710 (21·35%)	10·40 (4·44)	(1·98, 17·34)
ASD (age at dx)	2362 (54·46%)	111 (4·70%)	4·1 (3·99)[a]	(1, 17)
ADHD (age at dx)	2386 (55·01%)	342 (14·33%)	6·75 (3·40)[b]	(1, 16)
Allergy (age at screening)	1947 (44·89%)	853 (43·81%)	6·69 (4·31)	(0·15, 17·9)

Note. ADHD, attention-deficit/hyperactivity disorder; ASD, autism spectrum disorder; dx, diagnosis.

a only 84/111 (75.68%) of ASD subjects reported age

b only 261/342 (76.32%) of ADHD subjects reported age

Table S5: Full model results from the logistic regression model testing the association between maternal childhood maltreatment and child internalizing problems

	Model											
	2=Adjusted			3=CM*sex interaction			4=Maternal outcome			5=All		
Predictor	OR	95% CI	p	OR	95% CI	p	OR	95% CI	p	OR	95% CI	p
CM (No=ref)	4.04	(2.56, 6.38)	<0.0001	2.70	(1.95, 3.72)	<0.0001	2.37	(1.54, 3.64)	<0.0001	2.29	(1.64, 3.21)	<0.0001
Child sex (Male=ref)												
Female	0.91	(0.67, 1.23)	0.52	0.75	(0.45, 1.26)	0.28	0.90	(0.66, 1.22)	0.49	0.75	(0.44, 1.25)	0.26
Child race (White=ref)												
Black	0.79	(0.53, 1.19)	0.26	0.80	(0.54, 1.20)	0.28	0.88	(0.58, 1.32)	0.53	0.88	(0.59, 1.33)	0.55
Multiple Race	0.97	(0.60, 1.56)	0.90	0.98	(0.61, 1.57)	0.92	1.00	(0.62, 1.60)	0.99	1.00	(0.62, 1.60)	0.99
Other Race	1.42	(0.80, 2.53)	0.23	1.43	(0.80, 2.56)	0.23	1.55	(0.86, 2.79)	0.14	1.54	(0.86, 2.78)	0.15
Child ethnicity (Non-Hispanic=ref)												
Hispanic	0.89	(0.61, 1.29)	0.53	0.89	(0.61, 1.30)	0.56	0.94	(0.64, 1.36)	0.73	0.93	(0.64, 1.36)	0.72
Maternal age	0.96	(0.93, 1.00)	0.07	0.96	(0.93, 1.00)	0.07	0.97	(0.93, 1.02)	0.21	0.97	(0.93, 1.02)	0.21
Paternal age	1.04	(1.01, 1.07)	0.01	1.04	(1.01, 1.07)	0.01	1.03	(1.00, 1.07)	0.06	1.03	(1.00, 1.07)	0.06
Maternal education (college or more, some college, trade school, or college degree=ref)												

<High school	1.78	(1.10, 2.88)	0.02	1.80	(1.11, 2.93)	0.02	1.79	(1.12, 2.87)	0.02	1.80	(1.12, 2.90)	0.01
High school or GED	0.97	(0.60, 1.56)	0.90	0.97	(0.60, 1.56)	0.90	0.96	(0.59, 1.55)	0.87	0.96	(0.59, 1.55)	0.86
Income ($75k=ref)												
< $30k	2.00	(1.21, 3.29)	0.007	2.00	(1.21, 3.29)	0.007	1.85	(1.06, 3.20)	0.03	1.85	(1.07, 3.21)	0.03
$30k-50k	1.90	(1.15, 3.12)	0.01	1.89	(1.15, 3.12)	0.01	1.74	(0.99, 3.06)	0.05	1.74	(0.99, 3.06)	0.05
$50k-75k+	1.17	(0.67, 2.04)	0.59	1.17	(0.66, 2.05)	0.59	1.16	(0.65, 2.09)	0.61	1.17	(0.65, 2.09)	0.60
Maternal Depression (Yes=ref)							0.56	(0.39, 0.80)	0.002	0.56	(0.39, 0.80)	0.002
CM*female sex				1.33	(0.70, 2.52)	0.38				1.34	(0.70, 2.54)	0.37

Note. Model 2 is adjusted for all covariates except maternal diagnosis, model 3 is adjusted for all covariates except maternal diagnosis and includes the CM*sex interaction term, model 4 includes all covariates, model 5 includes all covariates and the CM*sex interaction term; CM, childhood maltreatment; ref, reference category, GED, general educational development; OR, odds ratio; CI, confidence interval.

Table S6: Full model results from the logistic regression model testing the association between maternal childhood maltreatment and child asthma

	Model											
	2=Adjusted			3=CM*sex interaction			4=Maternal outcome			5=All		
Predictor	OR	95% CI	p	OR	95% CI	p	OR	95% CI	p	OR	95% CI	p
CM (No=ref)	1.29	(1.06, 1.58)	0.01	1.54	(1.34, 1.77)	<0.0001	1.52	(1.26, 1.84)	<0.0001	1.46	(1.27, 1.67)	<0.0001
Child sex (Male=ref)												

Female	0.94	(0.82, 1.08)	0.41	0.93	(0.77, 1.12)	0.46	0.93	(0.81, 1.06)	0.28	0.93	(0.77, 1.12)	0.44
Child race (White=ref)												
Black	1.48	(1.24, 1.76)	<0.0001	1.48	(1.24, 1.76)	<0.0001	1.48	(1.24, 1.76)	<0.0001	1.48	(1.24, 1.76)	<0.0001
Multiple Race	1.16	(0.93, 1.43)	0.19	1.16	(0.93, 1.44)	0.19	1.18	(0.95, 1.46)	0.12	1.18	(0.95, 1.46)	0.13
Other Race	0.88	(0.65, 1.19)	0.42	0.88	(0.65, 1.19)	0.41	1.00	(0.75, 1.33)	0.98	1.00	(0.75, 1.33)	0.98
Child ethnicity (Non-Hispanic=ref)												
Hispanic	0.82	(0.70, 0.96)	0.02	0.82	(0.70, 0.96)	0.02	0.81	(0.69, 0.95)	0.01	0.81	(0.69, 0.95)	0.01
Maternal age	1.00	(0.98, 1.02)	0.97	1.00	(0.98, 1.02)	0.98	1.00	(0.98, 1.02)	0.87	1.00	(0.98, 1.02)	0.86
Paternal age	1.00	(0.99, 1.02)	0.69	1.00	(0.99, 1.02)	0.70	1.00	(0.99, 1.02)	0.70	1.00	(0.99, 1.02)	0.72
Maternal education (college or more, some college, trade school, or college degree=ref)												
<High school	1.08	(0.83, 1.39)	0.57	1.08	(0.84, 1.39)	0.56	1.11	(0.83, 1.48)	0.48	1.11	(0.83, 1.48)	0.48
High school or GED	0.96	(0.77, 1.20)	0.72	0.96	(0.76, 1.20)	0.69	0.98	(0.79, 1.22)	0.89	0.98	(0.79, 1.22)	0.88
Income ($75k=ref)												
< $30k	1.44	(1.16, 1.80)	0.001	1.44	(1.16, 1.79)	0.001	1.41	(1.15, 1.73)	0.001	1.41	(1.15, 1.73)	0.001
$30k-50k	1.23	(0.97, 1.55)	0.09	1.22	(0.97, 1.55)	0.09	1.19	(0.91, 1.54)	0.20	1.19	(0.92, 1.54)	0.19

$50k-75k+	1.03	(0.82, 1.31)	0.79	1.04	(0.82, 1.31)	0.77	1.02	(0.79, 1.31)	0.89	1.01	(0.79, 1.31)	0.91
Maternal Asthma (Yes=ref)							0.54	(0.45, 0.63)	<0.0001	0.54	(0.46, 0.63)	<0.0001
CM*female sex				1.03	(0.78, 1.35)	0.84				0.99	(0.76, 1.30)	0.96

Note. Model 2 is adjusted for all covariates except maternal diagnosis, model 3 is adjusted for all covariates except maternal diagnosis and includes the CM*sex interaction term, model 4 includes all covariates, model 5 includes all covariates and the CM*sex interaction term; CM, childhood maltreatment; ref, reference category, GED, general educational development; OR, odds ratio; CI, confidence interval.

Table S7: Full model results from the logistic regression model testing the association between maternal childhood maltreatment and child obesity

	Model											
	2=Adjusted			3=CM*sex interaction			4=Maternal outcome			5=All		
Predictor	OR	95% CI	p	OR	95% CI	p	OR	95% CI	p	OR	95% CI	p
CM (No=ref)	1.14	(0.89, 1.45)	0.31	1.11	(0.92, 1.34)	0.27	0.87	(0.68, 1.12)	0.29	1.12	(0.93, 1.35)	0.24
Child sex (Male=ref)												
Female	0.86	(0.72, 1.03)	0.10	0.66	(0.50, 0.85)	0.002	0.85	(0.71, 1.02)	0.08	0.64	(0.49, 0.83)	0.001
Child race (White=ref)												
Black	1.55	(1.24, 1.95)	0.0001	1.56	(1.24, 1.95)	0.0001	1.50	(1.20, 1.88)	0.0004	1.50	(1.20, 1.88)	0.0004
Multiple Race	1.25	(0.92, 1.69)	0.15	1.25	(0.92, 1.68)	0.15	1.20	(0.89, 1.62)	0.24	1.20	(0.89, 1.62)	0.24
Other Race	1.13	(0.74, 1.74)	0.57	1.12	(0.72, 1.72)	0.62	1.14	(0.74, 1.76)	0.56	1.12	(0.72, 1.73)	0.61
Child ethnicity												

(Non-Hispanic=ref)												
Hispanic	1.40	(1.11, 1.77)	0.005	1.42	(1.12, 1.79)	0.004	1.36	(1.07, 1.72)	0.01	1.37	(1.08, 1.74)	0.01
Maternal age	1.01	(0.98, 1.03)	0.59	1.01	(0.98, 1.03)	0.61	1.00	(0.98, 1.03)	0.72	1.00	(0.98, 1.03)	0.73
Paternal age	0.99	(0.97, 1.02)	0.56	0.99	(0.97, 1.02)	0.61	0.99	(0.97, 1.02)	0.56	0.99	(0.97, 1.02)	0.62
Maternal education (college or more, some college, trade school, or college degree=ref)												
<High school	1.37	(0.94, 1.98)	0.10	1.36	(0.94, 1.98)	0.10	1.35	(0.96, 1.89)	0.08	1.34	(0.96, 1.88)	0.09
High school or GED	1.06	(0.78, 1.43)	0.72	1.05	(0.78, 1.42)	0.73	1.02	(0.74, 1.40)	0.92	1.01	(0.74, 1.40)	0.93
Income ($75k=ref)												
< $30k	1.65	(1.22, 2.22)	0.001	1.65	(1.23, 2.23)	0.001	1.63	(1.22, 2.18)	0.001	1.64	(1.22, 2.19)	0.0009
$30k-50k	2.05	(1.50, 2.78)	<0.0001	2.04	(1.50, 2.78)	<0.0001	1.98	(1.46, 2.69)	<0.0001	1.99	(1.47, 2.69)	<0.0001
$50k-75k+	1.54	(1.11, 2.14)	0.009	1.56	(1.12, 2.15)	0.008	1.50	(1.09, 2.08)	0.01	1.52	(1.10, 2.11)	0.01
Maternal Obesity (Yes=ref)							0.59	(0.46, 0.76)	<0.0001	0.59	(0.46, 0.75)	<0.0001
CM*female sex				1.69	(1.17, 2.44)	0.005				1.75	(1.21, 2.53)	0.003

Note. Model 2 is adjusted for all covariates except maternal diagnosis, model 3 is adjusted for all covariates except maternal diagnosis and includes the CM*sex interaction term, model 4 includes all covariates, model 5 includes all covariates and the CM*sex interaction term; CM, childhood maltreatment; ref, reference category, GED, general educational development; OR, odds ratio; CI, confidence interval.

Table S8: Full model results from the logistic regression model testing the association between maternal childhood maltreatment and child ADHD

	Model											
	2=Adjusted			3=CM*sex interaction			4=Maternal outcome			5=All		
Predictor	OR	95% CI	p	OR	95% CI	p	OR	95% CI	p	OR	95% CI	p
CM (No=ref)	2.31	(1.70, 3.15)	<0.0001	2.09	(1.63, 2.67)	<0.0001	2.01	(1.49, 2.70)	<0.0001	2.07	(1.61, 2.65)	<0.0001
Child sex (Male=ref)												
Female	0.37	(0.28, 0.48)	<0.0001	0.34	(0.23, 0.51)	<0.0001	0.36	(0.28, 0.47)	<0.0001	0.34	(0.23, 0.51)	<0.0001
Child race (White=ref)												
Black	0.91	(0.67, 1.23)	0.53	0.91	(0.67, 1.23)	0.53	0.95	(0.70, 1.29)	0.73	0.95	(0.70, 1.29)	0.73
Multiple Race	0.48	(0.29, 0.79)	0.004	0.48	(0.29, 0.79)	0.004	0.46	(0.28, 0.76)	0.003	0.46	(0.28, 0.76)	0.003
Other Race	0.17	(0.05, 0.54)	0.003	0.17	(0.05, 0.54)	0.003	0.17	(0.05, 0.55)	0.003	0.17	(0.05, 0.55)	0.003
Child ethnicity (Non-Hispanic=ref)												
Hispanic	0.78	(0.54, 1.11)	0.17	0.78	(0.54, 1.11)	0.17	0.80	(0.56, 1.14)	0.21	0.80	(0.56, 1.14)	0.22
Maternal age	0.98	(0.95, 1.02)	0.31	0.98	(0.95, 1.02)	0.31	0.99	(0.96, 1.02)	0.45	0.99	(0.96, 1.02)	0.45
Paternal age	0.99	(0.96, 1.02)	0.45	0.99	(0.96, 1.02)	0.46	0.99	(0.96, 1.01)	0.36	0.99	(0.96, 1.01)	0.36
Maternal education (college or more, some college, trade school, or college degree=ref)												

<High school	1.17	(0.75, 1.84)	0.48	1.17	(0.75, 1.84)	0.49	1.16	(0.74, 1.81)	0.52	1.16	(0.74, 1.81)	0.52
High school or GED	0.89	(0.59, 1.34)	0.58	0.89	(0.59, 1.34)	0.58	0.89	(0.59, 1.32)	0.55	0.88	(0.59, 1.32)	0.55
Income ($75k=ref)												
< $30k	1.51	(1.02, 2.24)	0.04	1.51	(1.02, 2.24)	0.04	1.44	(0.95, 2.18)	0.09	1.44	(0.95, 2.19)	0.09
$30k-50k	1.40	(0.94, 2.11)	0.10	1.41	(0.94, 2.11)	0.10	1.35	(0.91, 2.00)	0.14	1.35	(0.91, 2.00)	0.14
$50k-75k+	1.43	(0.97, 2.10)	0.07	1.43	(0.97, 2.10)	0.07	1.46	(1.00, 2.14)	0.05	1.46	(1.00, 2.14)	0.05
Maternal ADHD (Yes=ref)							0.56	(0.36, 0.88)	0.01	0.56	(0.36, 0.88)	0.01
CM*female sex				1.13	(0.67, 1.92)	0.64				1.11	(0.65, 1.87)	0.71

Note. Model 2 is adjusted for all covariates except maternal diagnosis, model 3 is adjusted for all covariates except maternal diagnosis and includes the CM*sex interaction term, model 4 includes all covariates, model 5 includes all covariates and the CM*sex interaction term; CM, childhood maltreatment; ref, reference category, GED, general educational development; OR, odds ratio; CI, confidence interval.

Table S9: Full model results from the logistic regression model testing the association between maternal childhood maltreatment and child ASD

	Model											
	2=Adjusted			3=CM*sex interaction			4=Maternal outcome			5=All		
Predictor	OR	95% CI	p	OR	95% CI	p	OR	95% CI	p	OR	95% CI	p
CM (No=ref)	1.70	(1.13, 2.55)	0.01	1.89	(1.17, 3.04)	0.009	--	--	--	--	--	--
Child sex (Male=ref)												

Female	0.30	(0.19, 0.47)	<0.0001	0.37	(0.19, 0.72)	0.003	--	--	--	--	--	--
Child race (White=ref)												
Black	0.70	(0.39, 1.27)	0.24	0.70	(0.39, 1.27)	0.24	--	--	--	--	--	--
Multiple Race	0.70	(0.35, 1.41)	0.32	0.71	(0.35, 1.43)	0.33	--	--	--	--	--	--
Other Race	0.85	(0.36, 1.98)	0.70	0.85	(0.36, 1.98)	0.71	--	--	--	--	--	--
Child ethnicity (Non-Hispanic=ref)												
Hispanic	2.29	(1.44, 3.65)	0.0005	2.28	(1.43, 3.63)	0.0005	--	--	--	--	--	--
Maternal age	0.98	(0.93, 1.03)	0.34	0.98	(0.93, 1.03)	0.36	--	--	--	--	--	--
Paternal age	1.04	(1.00, 1.08)	0.08	1.03	(1.00, 1.08)	0.08	--	--	--	--	--	--
Maternal education (college or more, some college, trade school, or college degree=ref)												
<High school	0.49	(0.18, 1.38)	0.18	0.49	(0.18, 1.38)	0.18	--	--	--	--	--	--
High school or GED	1.52	(0.85, 2.73)	0.16	1.53	(0.85, 2.75)	0.16	--	--	--	--	--	--
Income ($75k=ref)												
< $30k	1.19	(0.61, 2.32)	0.62	1.18	(0.60, 2.31)	0.63	--	--	--	--	--	--
$30k-50k	1.39	(0.75, 2.60)	0.30	1.39	(0.74, 2.59)	0.30	--	--	--	--	--	--

$50k-75k+	0.98	(0.47, 2.02)	0.95	0.97	(0.47, 2.01)	0.94	--	--	--	--	--	--
Maternal ASD (Yes=ref)							--	--	--	--	--	--
CM*female sex				0.66	(0.26, 1.66)	0.38				--	--	--

Note. Model 2 is adjusted for all covariates except maternal diagnosis, model 3 is adjusted for all covariates except maternal diagnosis and includes the CM*sex interaction term, models 4 and 5 are missing due to lack of variation in the maternal diagnosis variable; CM, childhood maltreatment; ref, reference category, GED, general educational development; OR, odds ratio; CI, confidence interval.

Table S10: Full model results from the logistic regression model testing the association between maternal childhood maltreatment and child allergy

	Model											
	2=Adjusted			3=CM*sex interaction			4=Maternal outcome			5=All		
Predictor	OR	95% CI	p	OR	95% CI	p	OR	95% CI	p	OR	95% CI	p
CM (No=ref)	1.11	(0.85, 1.46)	0.44	0.87	(0.72, 1.05)	0.15	0.86	(0.66, 1.11)	0.25	0.78	(0.63, 0.95)	0.02
Child sex (Male=ref)												
Female	0.88	(0.73, 1.06)	0.17	0.87	(0.67, 1.11)	0.26	0.82	(0.67, 1.00)	0.05	0.84	(0.64, 1.11)	0.22
Child race (White=ref)												
Black	1.21	(0.94, 1.56)	0.13	1.21	(0.94, 1.56)	0.13	1.11	(0.85, 1.45)	0.43	1.11	(0.85, 1.45)	0.43
Multiple Race	1.32	(0.99, 1.75)	0.06	1.32	(0.99, 1.75)	0.05	1.37	(1.01, 1.86)	0.04	1.37	(1.01, 1.86)	0.04
Other Race	0.94	(0.63, 1.39)	0.76	0.94	(0.63, 1.39)	0.76	0.89	(0.59, 1.33)	0.56	0.89	(0.59, 1.33)	0.56
Child ethnicity												

(Non-Hispanic=ref)												
Hispanic	1.45	(1.16, 1.81)	0.001	1.45	(1.16, 1.81)	0.001	1.38	(1.09, 1.73)	0.007	1.37	(1.09, 1.73)	0.007
Maternal age	1.01	(0.99, 1.04)	0.41	1.01	(0.99, 1.04)	0.41	1.02	(0.99, 1.06)	0.12	1.02	(0.99, 1.06)	0.12
Paternal age	1.00	(0.98, 1.02)	0.90	1.00	(0.98, 1.02)	0.90	1.00	(0.97, 1.02)	0.84	1.00	(0.97, 1.02)	0.84
Maternal education (college or more, some college, trade school, or college degree=ref)												
<High school	1.13	(0.78, 1.64)	0.51	1.13	(0.78, 1.64)	0.51	1.15	(0.80, 1.66)	0.45	1.15	(0.80, 1.66)	0.44
High school or GED	0.80	(0.60, 1.07)	0.13	0.80	(0.60, 1.07)	0.13	0.81	(0.58, 1.13)	0.22	0.81	(0.58, 1.13)	0.22
Income ($75k=ref)												
< $30k	0.74	(0.55, 0.99)	0.04	0.74	(0.55, 0.99)	0.04	0.73	(0.52, 1.04)	0.08	0.73	(0.52, 1.04)	0.08
$30k-50k	0.98	(0.70, 1.36)	0.89	0.98	(0.70, 1.36)	0.89	0.88	(0.62, 1.25)	0.47	0.88	(0.62, 1.24)	0.47
$50k-75k+	0.95	(0.66, 1.35)	0.76	0.95	(0.66, 1.35)	0.76	0.84	(0.58, 1.21)	0.35	0.84	(0.58, 1.21)	0.34
Maternal Allergy (Yes=ref)							0.85	(0.61, 1.18)	0.33	0.85	(0.61, 1.18)	0.33
CM*female sex				1.03	(0.72, 1.49)	0.87				0.94	(0.64, 1.40)	0.77

Note. Model 2 is adjusted for all covariates except maternal diagnosis, model 3 is adjusted for all covariates except maternal diagnosis and includes the CM*sex interaction term, model 4 includes all covariates, model 5 includes all covariates and the CM*sex interaction term; CM, childhood maltreatment; ref, reference category, GED, general educational development; OR, odds ratio; CI, confidence interval.

Table S11: Fit statistics for child health outcomes latent class analysis

	LL value	BIC	Entropy	LMR value, p-value
1 class	-8299.29	16629.77	na	na
2 class	-8214.90	16538.67	0.59	165.96, p<.001
3 class	-8192.56	16489.07	0.74	43.92, p<.001
4 class	-8184.37	16509.07	0.86	16.10, p=0.09
5 class	-8178.28	16533.27	0.66	11.98, p=0.34

Note. na=Not Applicable; LL=log likelihood; BIC=Bayesian Information Criterion; LMR= Lo-Mendell-Rubin likelihood ratio test.

Table S12: Fit statistics for maternal childhood maltreatment subtypes latent class analysis

	LL value	BIC	Entropy	LMR value, p-value
1 class	-9204.88	18435.75	na	na
2 class	-7227.74	14732.65	0.84	3661.42, p<.001
3 class	-7276.25	14640.86	0.78	120.58 p<.001
4 class	-7234.01	14587.56	0.73	82.83, p<.001
5 class	-7229.39	14609.50	0.78	9.06, p=0.12

Note. na=Not Applicable; LL=log likelihood; BIC=Bayesian Information Criterion; LMR= Lo-Mendell-Rubin likelihood ratio test.

Table S13. Full model results from the adjusted logistic regression model testing the association between maternal childhood maltreatment and latent classes of child health outcomes

	Moderate global risk v. asthma & allergy	Allergy/low risk v. asthma & allergy	Moderate global risk v. allergy/low risk
	OR (95% CI), p-value	OR (95% CI), p-value	OR (95% CI), p-value
CM	3.77 (2.029, 7.028), p<0.001	0.959 (0.774, 1.188), p=0.702	3.938 (2.263, 6.853), p<0.001
Hispanic	1.025 (0.474, 2.216), p=0.950	1.214 (0.956, 1.542), p=0.111	0.844 (0.430, 1.657), p=0.623
Education (college or more=ref)			
< High school	0.720 (0.226, 2.292), p=0.579	0.282, (0.251, 0.583), p<0.001	1.882 (0.672, 5.272), p=0.229
High school or GED	2.275 (0.960, 5.292), p=0.062	0.805 (0.559, 1.160), p=0.244	2.826 (1.343, 5.947), p=0.006
Race (White=ref)			

Black	0.701 (0.286, 1.716), p=0.437	0.833 (0.628, 1.106), p=0.206	0.841 (0.382, 1.851), p=0.667
More than 1 race	0.441 (0.122, 1.590), p=0.211	0.845 (0.615, 1.162), p=0.301	0.522 (0.159, 1.714), p=0.284
Other	0.320 (0.044, 2.352), p=0.263	0.789 (0.536, 1.163), p=0.230	0.406 (0.061, 2.707), 0.352
Income ($75k=ref)			
<$30K	1.239 (0.432, 3.551), p=0.690	1.004 (0.847, 1.903), p=0.981	1.234 (0.497, 3.061), p=0.650
$30K - $50K	1.993 (0.650, 6.117), p=0.228	1.270 (0.847, 1.903), p=0.247	1.570 (0.621, 3.969), p=0.341
$50K - $75K	1.410 (0.503, 3.955), p=0.514	1.238 (0.868, 1.765), p=0.239	1.139 (0.470, 2.763), p=0.773
Sex (female)	0.574 (0.297, 1.106), p=0.097	1.036 (0.843, 1.272), p=0.738	0.554 (0.311, 0.987), p=0.045
Maternal age	1.047 (0.953, 1.150), p=0.342	1.002 (0.977, 1.028), p=0.848	1.044 (0.960, 1.049), p=0.314
Paternal age	0.972 (0.901, 1.048), p=0.458	0.991 (0.970, 1.012), p=0.377	0.981 (0.918, 1.049), p=0.574

Note. CM, childhood maltreatment; ref, reference category; GED, general educational development; OR, odds ratio; CI, confidence interval.

Figure S1. Flow diagram depicting eligibility and size of the analytic sample

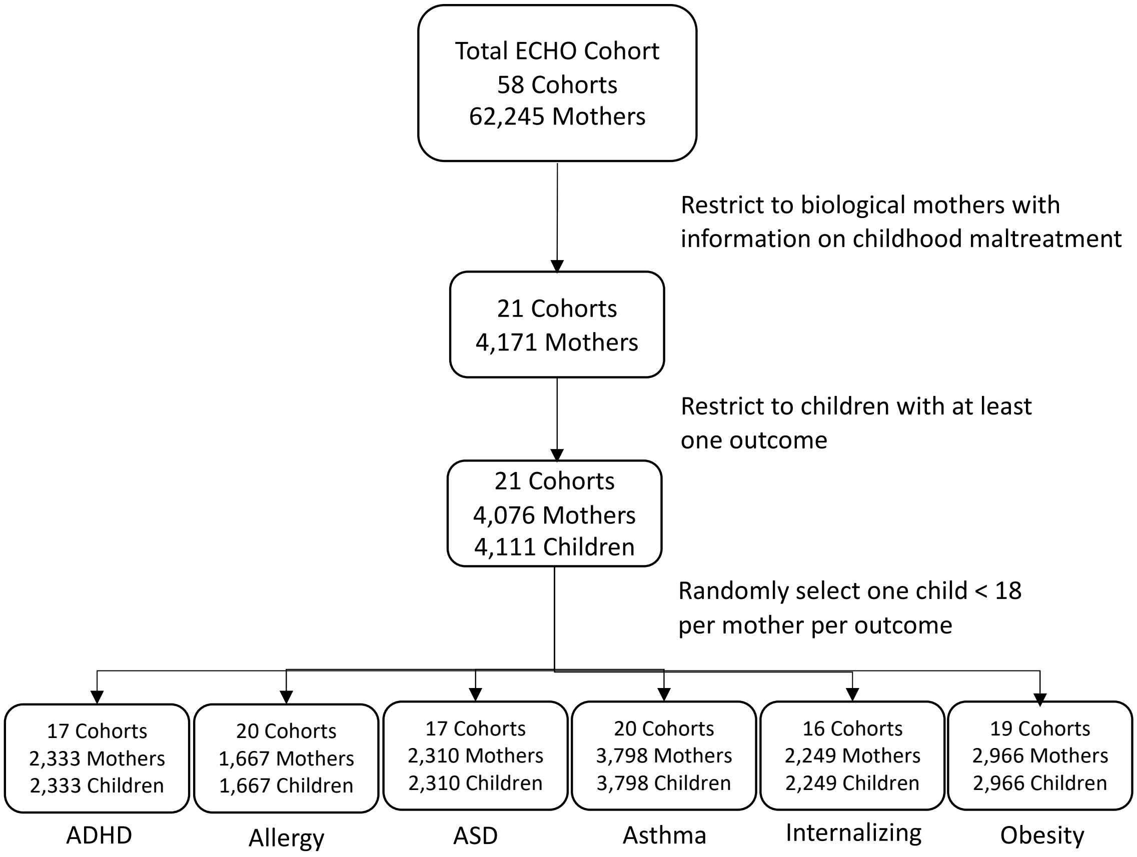

ECHO Collaborators Acknowledgement

The authors wish to thank our ECHO colleagues; the medical, nursing, and program staff; and the children and families participating in the ECHO cohorts. We also acknowledge the contribution of the following ECHO program collaborators:

ECHO Components—*Coordinating Center*: Duke Clinical Research Institute, Durham, North Carolina: Smith PB, Newby KL; *Data Analysis Center*: Johns Hopkins University Bloomberg School of Public Health, Baltimore, Maryland: Jacobson LP; Research Triangle Institute, Durham, North Carolina: Catellier DJ; *Person-Reported Outcomes Core*: Northwestern University, Evanston, Illinois: Gershon R, Cella D.

ECHO Awardees and Cohorts—Icahn School of Medicine at Mount Sinai, New York, NY: Teitelbaum SL; Stroustrup A; Cohen Children's Medical Center, Northwell Health: Stroustrup A; Cincinnati Children's Hospital Medical Center, Cincinnati, Ohio: Merhar S; Children's Hospital and Clinic Minnesota, Minneapolis, MN: Lampland A; University of Buffalo, Buffalo, NY: Reynolds A; University of Florida, College of Medicine, Jacksonville, FL: Hudak M; University of Rochester Medical Center, Rochester, NY: Pryhuber G; Vanderbilt Children's Hospital, Nashville, TN: Moore P; Pennsylvania State University, University Park, PA; Gatzke-Kopp; University of North Carolina, Chapel Hill, NC: Swingler M; University of Puerto Rico, San Jaun, PR: Canino G; Kaiser Permanente Northern California Division of Research, Oakland, CA: Croen L; University of Wisconsin, Madison WI: Gern J; Boston Medical Center, Boston MA: Bacharier L; Children's Hospital of New York: New York, NY: Bacharier L; Kattan M; Johns Hopkins University, School of Medicine, Baltimore, MD: Wood R; Bacharier L; Washington University in St Louis, St Louis, MO: Rivera-Spoljaric K; University of Southern California, Los Angeles, CA: Gilliland F; Farzan S; George Washington University, Washington, DC: Ganiban J; Pennsylvania State University, University Park, PA: Neiderhiser J; Oregon Health and Science University, Portland, OR: McEvoy C; Indiana University, Riley Hospital for Children: Indianapolis, IN,

Tepper R; Pennsylvania State University, University Park, PA: Lyall K; University of California, UC Davis Medical Center Mind Institute, Sacramento, CA: Ozonoff, S; University of Washington: Dager S; Children's Hospital of Philadelphia - Center for Autism Research: Schultz R; University of North Carolina at Chapel Hill: Piven J; Johns Hopkins Bloomberg School of Public Health: Volk H.

References

1. Nylund KL, Asparouhov T, Muthén BO. Deciding on the Number of Classes in Latent Class Analysis and Growth Mixture Modeling: A Monte Carlo Simulation Study. Structural Equation Modeling: A Multidisciplinary Journal. 2007 Oct 23;14(4):535–69.

2. Nylund-Gibson K, Masyn KE. Covariates and Mixture Modeling: Results of a Simulation Study Exploring the Impact of Misspecified Effects on Class Enumeration. Structural Equation Modeling: A Multidisciplinary Journal. 2016 Nov 1;23(6):782–97.

3. Nylund-Gibson K, Grimm RP, Masyn KE. Prediction from Latent Classes: A Demonstration of Different Approaches to Include Distal Outcomes in Mixture Models. Structural Equation Modeling: A Multidisciplinary Journal. 2019 Nov 2;26(6):967–85.

4. Little TD, Jorgensen TD, Lang KM, Moore EWG. On the joys of missing data. J Pediatr Psychol. 2014 Mar;39(2):151–62.

5. Schafer JL, Graham JW. Missing data: our view of the state of the art. Psychol Methods. 2002 Jun;7(2):147–77.
